# Selected Predictors of the Importance Attached to Salt Content Information on the Food Packaging (a Study among Polish Consumers)

**DOI:** 10.3390/nu12020293

**Published:** 2020-01-22

**Authors:** Paweł Bryła

**Affiliations:** Department of International Marketing and Retailing, Faculty of International and Political Studies, University of Lodz, Narutowicza 59a, 90-131 Lodz, Poland; pawel.bryla@uni.lodz.pl; Tel.: +48-4266-55830

**Keywords:** salt information, salt content, salt label, sodium label, sodium information, nutritional information, nutritional labeling, salt information use, nutrition knowledge

## Abstract

This paper aims to identify selected antecedents of the importance attached to salt content information (ISCI) placed on food labels, on the basis of a representative survey of 1051 Polish consumers. The study was conducted with the use of the CAWI (Computer Assisted Web Interviews) method in 2018. Quota sampling was applied with reference to the following five criteria: sex, age, education, place of living (urban and rural areas), and region. In a multiple regression model, ISCI depends on the respondent’s: sex, age, evaluation of the quantity of nutrition claims, importance attached to nutrition claims, willingness to pay a price premium for products with nutrition claims, attention paid to health and nutrition claims, agreeing with the opinion that unreliable nutrition claims are a serious problem, evaluation of healthiness of one’s diet, self-rated knowledge about healthy nutrition, buying organic food, and reading front-of-package (FOP) labels during and after the purchase. The strongest effects on the importance attached to salt content information on the food packaging were displayed by the importance of nutrition claims, attention paid to nutrition and health claims, respondent’s age, FOP label reading at home, and agreeing that the use of unreliable nutrition claims is a serious problem.

## 1. Introduction

Consumer preferences for information vary widely and an optimal policy should provide different labels for different market segments. Increasing the amount of information may reduce its effectiveness among the low-income consumers it is intended to help [1]. Food consumers understand and value easily recognizable logos more than the information found on nutritional composition labels [2]. Front-of-package labels that include content descriptors are more effective in helping consumers to select lower-sodium products, and traffic light labels, which incorporate content descriptors and color coding, turned out most effective at helping participants select low-sodium products [3]. In a restaurant setting, traffic light and red stop sign warning labels significantly reduced sodium ordered compared with a control. Warning labels also increased knowledge about high sodium content [4]. More accurate use of the nutrition facts panel moderates the effect of product nutrition value on consumer evaluations [5]. Nutrition knowledge has a strong effect on general label use, degree of use, and on use of nutrient content [6]. A literature review demonstrated no consensus on the effect of age, income, or working status on nutritional label use. However, education and gender were found to positively affect label use. It also appears that consumers who are more concerned about nutrition and health are more likely to use nutritional labels. Consequently, consumers on a special diet, organic buyers, and those aware of the diet-disease relation are more likely to search for on-pack nutrition information than others [7]. Although consumers evaluate the nutrition table most positively, it receives little attention and does not stimulate healthy choices. Health goals of consumers increase attention to and use of nutrition labels, especially when these health goals concern specific nutrients [8]. In another study, age, education, income, household size, and nutrition knowledge had an impact on nutritional label use [9].

Excess sodium intake has an important, if not predominant, role in the pathogenesis of elevated blood pressure, one of the most important modifiable determinants of cardiovascular diseases. The strategies to reduce sodium intake include: (1) public education, (2) individual dietary counseling, (3) food labeling, (4) coordinated and voluntary industry sodium reduction, (5) government and private sector food procurement policies, and (6) regulations to modify sodium’s generally regarded as safe (GRAS) status [10]. Globally, the average daily dietary salt intake is more than double the recommended level. Key sources of salt in the diet include commercially prepared or manufactured food products and discretionary salt added by consumers during cooking and consumption. Therefore, a significant lowering in the current salt intake requires a shift in both commercial foods and consumer behavior [11]. Regan et al. [12] called for a multi-actor approach that utilizes co-designed, participatory tools to facilitate the involvement of all stakeholders, especially consumers, in making decisions around how best to achieve population-level salt reduction. In 2011, 10 countries had front-of-pack salt labelling schemes [13]. However, emphasizing salt reduction by means of a front-of-pack label can have a negative effect on taste perception and salt use [14]. It is worth noting salt labeling rules differ across countries, e.g., in Malaysia, only 62% of instant noodles displayed the salt content on their food label [15]. In my opinion, it may be advisable to consider some basic standards of salt labeling at the global level. Providing information about the salt content is necessary to encourage a healthy choice, but the claims placed on the packaging seem to be insufficient, as they contribute to the avoidance of the product. Increasing consumer awareness of nutrition claims of foods is required [16]. Consumers’ knowledge about their health is a precondition for changing related behavior, which entails objective knowledge about salt intake, sources of salt, and ultimately the salt information on food labels [17]. It is necessary to identify strategies to improve individuals’ health and nutrition literacy, to develop strategies that help consumers comprehend and apply Nutrition Facts Label information, and to assess the impact of label usage on dietary behaviors [18]. Smartphone apps may be effective in supporting people, especially with cardiovascular diseases, to make lower salt food purchases [19]. Culture-specific awareness campaigns on salt intake and its association with health are needed [20].

There is a large variation of salt content information use across countries and over time as well as according to certain socio-demographic criteria. In the UK, in 2005, 38% of respondents looked at labelling to find out salt content, and 33% said that salt content would always affect their decision to buy a product [21]. In New Zealand, most participants did not know how to interpret the nutritional information, and many underestimated the salt content of the product by confusing it with sodium content [22]. Similarly, in Japan, few people could convert sodium content to salt, which suggested difficulty in using food labels to control their salt intake [23]. In Melbourne, 69% of respondents reported reading the salt content of food products when shopping. Salt label usage was significantly related to shoppers concern about the amount of salt in their diet and the belief that their health could improve by lowering salt intake. Approximately half of the sample was unable to accurately use labelled sodium information to pick low salt options [24]. In 2007, 70% of Australian consumers correctly identified that most dietary salt comes from processed foods but only a quarter regularly checked food labels for salt content. Even fewer reported that their food purchases were influenced by the salt level indicated (21%) [25]. In a more recent study, conducted in 2015, 89% of Australians were aware of the health risks associated with a high salt intake, 75% correctly identified salt from processed foods as being the main source of salt in the diet, but only 28% could correctly identify the maximum recommended daily intake for salt [26]. Brazilian consumers were concerned about the amount of salt (sodium chloride) in the products they consumed, regardless of educational levels, income, age, lifestyles, or health conditions. The majority of respondents rarely read the sodium content on food labels; however, men and older individuals were more likely to read label information on sodium content [27]. In China, only 5% of respondents understood the meaning of NRV% (Percentage of Nutrient Reference Values), 48% did not know the relationship between sodium and salt, and 13% reported they frequently read the label when shopping. Factors for why people were more likely to choose a product because of its low level of salt shown on the label included income level and their level of awareness of the link between salt and diet [28]. In Korea, the proportion of female college students who read the nutrition information reached 62% but it was only 32% for the sodium information. Their intention to buy low sodium foods increased up to 40% if sodium information was provided on the food label [29]. In Lebanon, only 38% of respondents checked for salt label content, 44% reported that their food purchases were influenced by salt content, and 39% tried to buy low-salt foods [30]. Among Pakistani women, a relationship was found between knowledge about low-salt foods and using low-salt labels [31]. In Pakistan, people of upper castes, people in large families, respondents who were advised to lower salt intake, and who checked salt/sodium labels were less likely to consume higher amounts of salt [32]. In the United States, 19% of respondents agreed they were confused about how to figure out how much sodium is in the foods they eat, and 47% reported they check nutrition labels for sodium content as a tactic to limit salt. Consumers with a high school education or less were more likely than college graduates to report they were confused about sodium content on labels and less likely to check labels for sodium as a tactic to limit salt intake [33]. In Denmark, most consumers are willing to purchase salt-reduced food products, even without having a salt reduction goal. Personal and social norms reveal the strongest influences on intention to change dietary habits, whereas personal norms, knowledge, and awareness of health consequences exert the strongest influences on willingness to purchase salt-reduced food products [34]. In Poland, even commodity science students could not correctly interpret information provided by the Guideline Daily Amount (GDA) system, despite their declarations of full or partial understanding of nutritional labeling [35]. According to Polish food processors and distributors, the information on the content of salt was the third most important type of nutritional information on the food packaging, following content of sugar and of fat [36], whereas among Polish consumers, the information on the salt content ranked fourth after the content of sugar, vitamins, and fats [37]. However, the fact that information about salt content information is missing in some product categories (e.g., cereal products) makes consumer choice more difficult [38]. In a large-scale international survey in Germany, Austria, USA, Hungary, India, China, South Africa, and Brazil, it was found that while salt reduction was seen to be healthy and important, over one third of participants were not interested in salt reduction and the majority were unaware of recommendations [39]. A recent review of 24 studies across 12 countries showed that while consumers were aware of the health implications of a high salt intake, fundamental knowledge regarding recommended dietary intake, primary food sources, and the relationship between salt and sodium was lacking. Moreover, many participants were confused by nutrition information panels, but food purchasing behaviors were positively influenced by front of package labelling [40].

This paper aims to identify selected antecedents of the importance attached to salt content information (ISCI) placed on food labels, on the basis of a large-scale, representative survey of Polish consumers. To the best of my knowledge, this is the first study to investigate the ISCI in a representative, nation-wide sample of Polish consumers. As the literature review showed large differences across nations, it is necessary to examine this issue in the biggest Central European country. The age-standardized estimated sodium intake for persons aged 20 and over was at the level of 3.84 g/day in Poland in 2010, which was similar to the global average of 3.95 g/day [41]. However, within the European Union, Central European countries, including Poland, rank at the top of the table of estimated salt intakes. Poland ranked fifth in the EU with the average salt intake of as much as 11.5 g/day, and five out of the six countries with the highest consumption of salt per capita in the European Union were from the Central European region (Czech Republic, Slovenia, Hungary, Poland, and Romania), with the exception of Portugal taking the fourth place [42]. A wide range of potential predictors were analyzed to see if they differentiated the ISCI in a statistically significant way. Next, a multiple regression model was constructed to examine the simultaneous impact of various independent variables. Finally, a simplified model with only significant predictors was arrived at. The main contribution of this paper lies not only in studying the phenomenon in a new geographic context, but also identifying new predictors of the importance attached to salt content information, in particular concerning various aspects of the attitude to nutrition claims and the context of reading labels (at home rather than in the shop).

## 2. Materials and Methods

The study was conducted with the use of the CAWI (Computer Assisted Web Interviews) method in 2018. The online survey was administered by a specialized research agency commissioned by the University of Lodz. The author of this manuscript designed the questionnaire and set sampling criteria. The respondents were informed that the results would be used only for scientific purposes with the respect of the principle of anonymity. The sample size amounted to 1051 persons. Quota sampling was applied with reference to the following five criteria: sex (males and females), age (the following age intervals: 15–24, 25–34, 35–44, 45–54, 55–64 and 65 and more), education (primary, secondary, tertiary), place of living (urban and rural areas) and voivodeship (all 16 Polish regions). Thanks to this approach, the structure of the sample was similar to the general population of Polish consumers according to the aforementioned criteria.

The sample comprised of 560 women (53.3%) and 491 men (46.7%). Regarding the age structure, the sample was composed in the following way: 15–24 years—15.0%, 25–34—17.3%, 35–44—17.9%, 45–54—13.9%, 55–64—15.7%, 65 and more—20.2%. The mean age amounted to 45.0. As far as the household size is concerned, the structure was as follows: 1 person—9.5%, 2—31.7%, 3—24.6%, 4—19.1%, 5—7.7%, 6 and more—7.3%. Regarding the number of children in the household, the sample was structured in the following way: 0 children—52.4%, 1—25.1%, 2—16.9%, 3—3.8%, 4—1.2%, 5 and more—0.5%. The sample resembled the general population in terms of the education level, with 47.7% of respondents having primary and vocational education, 31.6%—secondary, and 20.7%—tertiary. Regarding professional activity, the sample had the following characteristics: white-collar workers—13.3%, blue-collar workers—28.0%, unemployed—4.7%, students—10.5%, not working and caring for the family—9.5%, old age pensioners and disability pensioners—29.7%. As far as the family monthly net income is concerned, the structure was as follows: under 2000 PLN—15.0%, 2001–3000—23.8%, 3001–4000—21.9%, 4001–5000—18.2%, 5001–6000—10.5%, over 6000—10.7%. 61.7% of the respondents lived in the urban areas, while 38.3% were rural inhabitants. Regarding the size of city, the structure was as follows: rural areas—38.3%, town up to 50,000 inhabitants—18.4%, city of 50,000–500,000—18.6%, city having more than 500,000 inhabitants—14.7%. All 16 Polish regions were represented in the sample, with the highest shares from the most populated regions—Mazowieckie (with the national capital Warsaw)—13.5% and Śląskie (Silesia)—11.4%.

The operationalization of the key variables used in this study is provided in Table 1.

In order to analyze the collected empirical material, *t*-tests, analyses of variance (ANOVAs), Pearson correlation coefficients, and multiple regression models were applied. The analyses were conducted in Statistica 12.0 (TIBCO Software Inc., Palo Alto, CA, USA).

## 3. Results

My dependent variable was the importance attached to salt content information on food packaging (ISCI). It was measured with the use of the following question: ‘How important is the following information on the packaging of food products?—Salt content’ with five answer options: very big, rather big, average, rather small, none, which were subsequently coded in the scale 5-1.

Sex differentiated the level of ISCI. Women attached higher importance to salt content information on food packaging than men (Mean: 3.896 versus 3.635, t = 4.134, *p* < 0.001).

Age correlated positively with the ISCI (r = 0.137, *p* < 0.001), meaning that older respondents declared a higher importance of this type of information.

The importance attached to salt content information on food packaging was analyzed with the use of ANOVAs based on selected characteristics of respondents: place of living (size of the city), household size, number of children in the household, respondent’s education level, occupational status, household income, evaluation of the quantity of nutrition claims, and the most important information during the first purchase of a food product (Table 2).

The place of living, understood as the size of the respondent’s city, did not affect in a significant way the importance attached to salt content information. The household size did not affect in a significant way the importance attached to salt content information.

The number of children in the respondent’s household marginally affected the importance attached to salt content information. Respondents from families with no children and 1 child attached higher importance to this kind of information compared to those with 2 or more children.

The importance attached to salt content information depends on the level of education. The higher the education, the more importance is attached to this kind of information.

Occupational status influenced the level of importance attached to salt content information. The highest importance was declared by pensioners (including old age pensioners and disability pensioners) and white-collar workers, whereas the lowest was displayed by students, which is at least partly related to the age structure of these groups.

Income marginally affected the importance attached to salt content information on food packaging. The highest importance was observed in the group of respondents with middle income—3001–4000 PLN (approximately 700–900 EUR) of total monthly disposable income of their household. The lowest importance of this kind of information was indicated by respondents with extremely low and high income.

The evaluation of the quantity of nutrition claims on food packaging affected the importance attached to salt content information significantly. Respondents feeling that there were too many nutrition claims attributed less importance to salt content information than those who believed that this quantity was appropriate or insufficient.

The ISCI depended on which information was the most important for respondents when they bought a food product for the first time. Price was deliberately excluded from the catalogue of answers, and the respondents were asked to consider only non-price attributes. It turned out that the highest ISCI characterized those who indicated nutrition information as the most important type of information during the first purchase.

Being on a special diet for health reasons significantly increased the declared importance of salt content information (4.006 versus 3.728, t = 3.271, *p* = 0.001).

Body Mass Index (BMI) correlated positively with the level of importance attached to salt content information (r = 0.378, *p* < 0.001), meaning that people with higher BMI paid more attention to the salt content information.

Self-rated health correlated strongly with the ISCI (r = 0.917, *p* < 0.001). Respondents evaluating their health more favorably attributed more importance to the salt content information.

The ISCI correlated strongly with understandability of nutrition claims (r = 0.913, *p* < 0.001), showing that understanding nutrition claims better increased the importance attached to salt content information.

There was a strong correlation of ISCI with the perceived credibility of nutrition claims (r = 0.928, *p* < 0.001), underlining the link between the importance attached to such information and trust in its accuracy.

The ISCI was strongly associated with the importance attached to nutrition claims on food packaging (r = 0.945, *p* < 0.001) as well as with the importance of other types of information placed on the packaging, such as: health claims (r = 0.944, *p* < 0.001), list of ingredients (r = 0.943, *p* < 0.001), expiry date (r = 0.932, *p* < 0.001), country of origin (r = 0.926, *p* < 0.001), culinary recipes (r = 0.914, *p* < 0.001), brand (r = 0.922, *p* < 0.001), organic label (r = 0.936, *p* < 0.001), quality signs (r = 0.938, *p* < 0.001), recommendations of scientific institutes (r = 0.930, *p* < 0.001), and price (r = 0.917, *p* < 0.001). As the importance of these types of information was highly correlated (correlation coefficients exceeding 0.9), I selected the importance attached to nutrition claims only for further analyses.

The ISCI was also strongly correlated with the importance ascribed to other types of nutrition information, such as: energy value (r = 0.954, *p* < 0.001), fat content (r = 0.968, *p* < 0.001), sugar content (r = 0.967, *p* < 0.001), protein content (r = 0.959, *p* < 0.001), vitamin content (r = 0.960, *p* < 0.001), roughage content (r = 0.961, *p* < 0.001), and Omega-3 fatty acids content (r = 0.961, *p* < 0.001). These results indicate that those attaching high importance to one type of nutrition information tend to appreciate highly also other types of such information. There are very strong correlations between these measures (correlation coefficients exceeding 0.95). Therefore, I excluded the importance attached to particular types of nutrition information from further analyses with the exception of the ISCI.

I also found strong correlations of the ISCI with the importance of all analyzed health claims, concerning the impact of the product on: lowering the cholesterol level (r = 0.942, *p* < 0.001), lowering the risk of heart diseases (r = 0.946, *p* < 0.001), strengthening bones (r = 0.947, *p* < 0.001), the digestive system (r = 0.948, *p* < 0.001), reducing tiredness and fatigue (r = 0.939, *p* < 0.001), maintaining proper vision (r = 0.943, *p* < 0.001), proper development of children (r = 0.929, *p* < 0.001), and proper functioning of the heart (r = 0.944, *p* < 0.001). Due to the very strong correlations among these variables (correlation coefficients above 0.9), they were not included in further analyses.

The ISCI was associated with the willingness to pay (WTP) a higher price for products with nutrition claims compared to similar products without such claims. The WTP was operationalized in two ways in my research. First, it was an answer to the question: ‘Are you willing to pay more for products with nutrition claims (compared to similar products without such claims)?’ The following answer options were proposed: definitely yes, rather yes, I don’t know, rather not, and definitely not, and subsequently coded in the scale 5-1. The second operationalization of the WTP was numerical. Those respondents who answered ‘definitely yes’ or ‘rather yes’ to the above question were asked to estimate how much higher a price (on average) they would be willing to pay for products with nutrition claims (compared to similar products without such claims), expressed in percentage. It turned out that the ISCI correlated significantly with both types of WTP. For the former, the correlation coefficient amounted to 0.925 (*p* < 0.001), whereas for the latter it was 0.152 (*p* = 0.001). The former measure of the WTP was selected for further analyses.

Unsurprisingly, the ISCI was strongly correlated to paying attention to health and nutrition claims (r = 0.945, *p* < 0.001).

More surprising was a very strong correlation between the ISCI and the opinion that the use of unreliable nutrition claims is a serious problem in Poland (r = 0.928, *p* < 0.001). Paradoxically, respondents who confirmed the existence of this problem attached a higher importance to salt content information. They might have a better knowledge of this negative phenomenon, which did not prevent them from paying attention to nutrition claims.

Consumers evaluating their diet as more healthy indicated a higher importance of salt content information on the packaging (r = 0.944, *p* < 0.001).

The ISCI was also related to the self-evaluated knowledge on a healthy diet (r = 0.941, *p* < 0.001).

Consumers purchasing certain types of products were characterized by significantly higher importance attached to salt content information (Table 3). It applied to: dietary supplements, organic food, functional food, and fair trade products.

The ISCI was significantly related to reading food labels, both during the purchase in the point of sale, and at home after the purchase (Table 4). It was correlated with front-of-package and back-of-package labels in both contexts. Please not that this variable was operationalized as the percentage share of products during and after the purchase of which the respondent reads information placed on the packaging. This measurement approach reduces potential bias between heavy buyers and those who do not engage in food shopping frequently, as I focus on the relative, not absolute frequency of reading labels.

Variables which significantly affected the importance attached to salt content information in the *t* tests, correlation coefficients, and ANOVAs (with the exception of those that were highly correlated with similar independent variables) were included in a multiple regression model in order to test their simultaneous impact on the dependent variable (ISCI) (Table 5). The initial regression model included 27 independent variables, 11 out of which turned out to be statistically significant predictors of the ISCI at the level of *p* < 0.05. The whole model was highly significant (*p* < 0.001) and explained 32.8% of the variance of the dependent variable.

In order to arrive at a more parsimonious model, I gradually eliminated from the full model those predictors that failed to reach statistical significance (*p* < 0.05), starting with those that had the highest statistical significance in the initial model. The implementation of this procedure led to the emergence of the final multiple regression model (Table 6). It explains almost the same amount of the variance of the dependent variable (R^2^ = 0.320, *p* < 0.001), with a considerably smaller set of predictors (12 variables). It is worth noting that all independent variables which were significant in the initial model remain significant in this modified model, and one variable which failed to reach statistical significance previously has now become significant (sex).

The importance attached to salt content information on food packaging depends on the respondent’s: sex, age, evaluation of the quantity of nutrition claims, importance attached to nutrition claims, willingness to pay a price premium for products with nutrition claims, attention paid to health and nutrition claims, agreeing with the opinion that unreliable nutrition claims are a serious problem, evaluation of healthiness of one’s diet, self-rated knowledge about healthy nutrition, buying organic food, and reading front-of-package labels during and after the purchase. Being a woman increases the ISCI. Older consumers attach more importance to salt content information. Evaluating the quantity of nutrition claims as appropriate and insufficient reinforces the ISCI. Higher importance attached to nutrition claims in general translates into a higher ISCI. Being more willing to pay a higher price for products with nutrition claims increases the ISCI. Paying more attention to health and nutrition claims increases the ISCI. Agreeing with the opinion that the use of unreliable nutrition claims constitutes an important problem boosts the ISCI. Evaluating one’s diet as healthy contributes to a higher ISCI. Displaying better (self-rated) knowledge about healthy nutrition also improves the ISCI. Buying organic food is positively related to the ISCI. A bit surprisingly, reading front-of-package information in the shop actually reduces the ISCI, whereas reading the same kind of information on the label after the purchase at home increases it.

The multivariate model can be used for profiling consumer characteristics more prone to value ISCI. I estimated the predicted ISCI for a sensitive set of combination of covariates in the regression model (Table 7). The predictions for different levels of a given independent variable were made on the assumption that the remaining covariates were at their mean level observed in the sample.

On the basis of these calculations, we can notice, for instance, that the difference in the predicted ISCI between men and women is 3.831 − 3.710 = 0.121. In relative terms, it is 0.121/3.710 = 0.0326, i.e., 3.26%. The ISCI values for men and women actually observed in the sample were 3.635 and 3.896 respectively, which means a difference between sexes of 0.261 or 7.18%. Does it mean that the above predictions are inaccurate? Not necessarily, because we should bear in mind that the multiple regression takes into account the simultaneous impact of all predictors included in the model. For example, if women in the sample tend to be younger than men and age is positively correlated with ISCI, the impact of age is not reflected in the ISCI ANOVA for sex, but it is taken into account in the multiple regression with ISCI as the dependent variable and both sex and age included as predictors. Ceteris paribus, being older by 10 years leads to a higher predicted ISCI by 0.091. Ceteris paribus, those who consider the quantity of nutrition claims on the food packaging as insufficient have a higher predicted ISCI than those who think there are too many nutrition claims by 0.272. Ceteris paribus, those who attach very high importance to nutrition claims have a predicted ISCI higher than those who consider their importance as none by as much as 1.092. Ceteris paribus, those who declare they are definitely willing to pay more for products with nutrition claims than for analogous products without such claims have their predicted ISCI higher by 0.279 than those who definitely would not pay more. Ceteris paribus, those who definitively pay attention to health and nutrition claims have their predicted ISCI higher than those who definitively do not by 0.725. Ceteris paribus, those who definitively agree with the statement that the use of unreliable nutrition claims is a serious problem have their predicted ISCI higher by 0.497 than those who definitively do not agree with this opinion. Ceteris paribus, those who consider their diet as very healthy have their predicted ISCI higher than those who consider it very unhealthy by 0.484. Ceteris paribus, those who evaluate their knowledge about healthy nutrition as very big have their predicted ISCI higher than those who think it is very small by 0.383. Ceteris paribus, buying organic food leads to an increase in the predicted ISCI by 0.117. Ceteris paribus, those who declare they read FOP labels for 90% of food products in the shop have their predicted ISCI lower by 0.255 than those who declare reading such labels for 10% of food products in the point of purchase. Ceteris paribus, those who declare reading 90% of FOP labels at home have their predicted ISCI higher by 0.298 than those who read only 10% of such labels after shopping.

## 4. Discussion

Only two out of the 12 statistically significant predictors of the importance attached to salt content information were demographic (sex and age), while the remaining 10 were behavioral and psychographic. The strongest effects on the importance attached to salt content information on the food packaging (|β| > 0.1) were displayed by the importance of nutrition claims, attention paid to nutrition and health claims, respondent’s age, FOP label reading at home, and agreeing that the use of unreliable nutrition claims is a serious problem. Therefore, front-of-package labeling plays a key role in salt content communication, which is congruent with references [2,3,4,39]. Second, this study confirmed the impact of gender, age, buying organic food [7], and consumer knowledge [6,34] on the nutritional label use. Contrary to some previous studies [9,33], education, income, and household size did not affect the importance attached to salt content information in the multiple regression model. Contrary to a study conducted in Brazil [27], not men, but women attached more importance to the salt information.

The contribution of the current study is related to the fact that it enabled to focus on the importance attached to salt content information (regardless of its form on the label), rather than nutritional information in general on the one hand, and specific nutrition claims on the other hand. Furthermore, my findings indicate the importance of several new predictors of the ISCI, including various aspects of the attitude to nutrition claims and the context of reading FOP labels (at home rather than in the shop). Third, it is the first attempt to study this phenomenon in a representative sample of the inhabitants of Poland, the largest country in the Central European region, which is characterized by very unfavorable salt intake statistics within the European Union.

There are a few implications of my findings. First of all, there is a need to increase the importance attached to salt content information in certain segments of the population, especially among men and younger consumers. Second, it is advisable to conduct education campaigns stressing the importance of nutrition claims placed on food products, explaining the meaning of nutrition information presented on the labels, and increasing consumer knowledge about healthy nutrition. Third, it is recommended to augment consumers’ attention to health and nutrition claims, e.g., by allocating a more prominent place on the packaging to this type of information, using bigger fonts of the typeface, using graphical symbols alongside textual information, and emphasizing them in other forms of marketing communications, in particular advertising. Fourth, it is recommended to run social marketing campaigns in favor of more healthy diets. Fifth, it is recommended to stimulate the development of the organic food market. Sixth, it may be beneficial to encourage consumers to read food labels after the purchase at home, e.g., by running loyalty programs requiring consumers to use promotion codes placed on the packaging. Seventh, the use of nutrition claims and other similar types of information on the food labels should be controlled by independent public authorities so as to minimize the concern about unreliable claims. Eighth, it may be advisable to consider the expansion of the obligation of salt content labeling to other food product categories.

It is worth noting that all measures used in this study were self-reported, rather than observed, which may be considered a limitation. Second, due to the intention-behavior gap, it is hard to translate the declarations of importance of particular information types into actual purchasing behaviors. However, our variable of interest was importance attached to salt content rather than just reading salt labels, which may be considered more accurate in predicting consumer behavior in my opinion.

Future research may focus on the perception of various types of salt content information by different segments of consumers. Second, structural equation modeling may be used to examine various paths of causal relationships, mediators, and moderators. Third, long-term purchasing data in consumer panels may be used to investigate the impact of socio-demographic variables on the preferences for low sodium products and the relationship between reading salt information, attaching importance to it, and implementing healthy diet practices.

## Figures and Tables

**Table 1:** Operationalization of variables included in the regression models.

Variable	Operationalization
ISCI	Importance attached to salt content information (ISCI) on food packaging: very high—5, rather high—4, average—3, rather small—2, none—1
Sex	Woman—1, man—0
Age	In years
BMI	Body Mass Index—the body mass divided by the square of the body height
Special diet	Being on a special diet for health reasons: yes—1, no—0
Self-rated health	How do you evaluate your health status? Very good—5, rather good—4, average—3, rather poor—2, very poor—1
Education	Primary—0, vocational—1, secondary—2, tertiary—3
White-collar worker	Yes—1, no—0
Pensioner	Old age or disability pensioner: yes—1, no—0
Children	The number of children in the respondent’s household
Quantity of nutrition claims	Evaluation of the quantity of nutrition claims on the packaging of food products: excessive—1, appropriate—2, insufficient—3
Understandability of nutrition claims	Very understandable—5, rather understandable—4, average—3, rather not understandable—2, completely not understandable—1
Importance of nutrition claims	Very big—5, rather big—4, average—3, rather small—2, none—1
Credibility of nutrition claims	Very credible—5, rather credible 4, average—3, rather not credible—2, definitely not credible—1
Nutrition information at first purchase	Indicating nutrition information as the most important type of information on the label (with the exception of price) when the respondent buys a food product for the first time (1 or 0)
Willingness to pay for nutrition claims	Willingness to pay a higher price for a product with nutrition claims compared to a similar product without such claims: definitely yes—5, rather yes—4, I don’t know—3, rather not—2, definitely not—1
Attention to health and nutrition claims	Do you pay attention to health and nutrition claims? Definitely yes—5, rather yes—4, hard to say—3, rather not—2, definitely not—1
Unreliable nutrition claims	Agreement with the opinion that the use of unreliable nutrition claims is a serious problem in Poland: definitely yes—5, rather yes—4, hard to say—3, rather not—2, definitely not—1
Diet healthiness evaluation	How do you evaluate your diet? Very healthy—5, rather healthy—4, average—3, rather unhealthy—2, very unhealthy—1
Knowledge about healthy nutrition	How do you evaluate your knowledge about healthy nutrition? Very big—5, rather big—4, average—3, rather small—2, very small—1
Dietary supplements	Buying dietary supplements: yes—1, no and don’t know—0
Organic food	Buying organic food: yes—1, no and don’t know—0
Functional food	Buying functional food: yes—1, no and don’t know—0
Fair trade products	Buying fair trade products: yes—1, no and don’t know—0
FOP label reading in the shop	The share of food products during the purchase of which the respondent reads the Front-of-Package (FOP) label (%)
BOP label reading in the shop	The share of food products during the purchase of which the respondent reads the Back-of-Package (BOP) label (%)
FOP label reading at home	The share of food products after the purchase of which the respondent reads the Front-of-Package (FOP) label (%)
BOP label reading at home	The share of food products after the purchase of which the respondent reads the Back-of-Package (BOP) label (%)

**Table 2:** The importance attached to salt content information on food packaging by selected characteristics of respondents (analyses of variance—ANOVAs).

Independent Variables	Groups	ISCI	ANOVAs
Place of living	Rural areas	3.799	F = 0.555, *p* = 0.645
	Town up to 50 thousand	3.731	
	City of 50–500 thousand	3.807	
	City of over 500 thousand	3.701	
Household size (1)	1	3.790	F = 0.664, *p* = 0.651
	2	3.826	
	3	3.795	
	4	3.697	
	5	3.779	
	6	3.612	
Number of children (2)	0	3.815	F = 2.278, *p* = 0.059
	1	3.833	
	2	3.624	
	3	3.625	
	4	3.308	
Level of education	Primary	3.605	**F = 4.619, *p* = 0.003**
	Vocational	3.691	
	Secondary	3.804	
	Tertiary	3.968	
Occupational status	White-collar worker	3.950	**F = 5.350, *p* < 0.001**
	Blue-collar worker	3.660	
	Unemployed	3.633	
	Student	3.555	
	Housekeeper	3.630	
	Pensioner	3.962	
Income (3)	Below 2000 PLN	3.690	F = 1.985, *p* = 0.078
	2001–3000 PLN	3.736	
	3001–4000 PLN	3.943	
	4001–5000 PLN	3.723	
	5001–6000 PLN	3.836	
	Over 6000 PLN	3.661	
Nutrition claims (4)	Excessive	3.305	**F = 17.550, *p* < 0.001**
	Appropriate	3.729	
	Insufficient	4.014	
Type of information (5)	Country of origin	3.808	**F = 4.763, *p* < 0.001**
	Nutrition information	3.984	
	Health information	3.864	
	List of ingredients	3.881	
	Expiry date	3.612	

Notes: (1) Households with over 6 members were not included in the ANOVA. They were represented by 2.7% of the respondents. (2) Households with over 4 children were not included in the ANOVA. They were represented by 0.5% of the respondents. (3) Average monthly disposable income of the respondent’s household. (4) Evaluation of the quantity of nutrition claims on food packaging. (5) The most important information on the label (excluding price) during the first purchase of a food product. Respondents indicating ‘other’ and ‘don’t know’ were excluded from the ANOVA. They accounted for 0.6% and 2.1% of the sample, respectively. Significant results indicated in bold.

**Table 3:** The importance attached to salt content information on food packaging by purchasing certain products (*t* tests).

Purchasing	Yes	No	t	*p*
Dietary supplements	3.855	3.699	2.451	0.014
Organic food	4.023	3.482	8.790	<0.001
Functional food	3.987	3.658	5.013	<0.001
Fair trade products	3.906	3.725	2.557	0.011

Note: the category ‘No’ includes those who answered ‘No’ and ‘Don’t know’.

**Table 4:** Correlation coefficients between the importance attached to salt content information on food packaging and reading labels (the share of food products for which the respondent reads FOP or BOP labels in the shop or at home).

Reading Labels		r	*p*
Shop	FOP	0.071	**0.022**
	BOP	0.118	**<0.001**
Home	FOP	0.104	**0.001**
	BOP	0.116	**<0.001**

Note: FOP—front-of-package labels, BOP—back-of-package labels.

**Table 5:** Selected predictors of the importance attached to salt content information on food packaging (the initial multiple regression model).

Predictors	β	SE	t	*p*
Intercept	x	x	0.421	0.674
Sex	0.052	0.028	1.872	0.062
Age	0.139	0.041	3.419	**0.001**
BMI	−0.027	0.030	−0.889	0.374
Special diet	0.007	0.028	0.248	0.804
Self-rated health	−0.034	0.031	−1.078	0.281
Education	0.014	0.031	0.464	0.643
White-collar worker	0.018	0.030	0.606	0.544
Pensioner	0.011	0.039	0.281	0.779
Children	−0.026	0.028	−0.940	0.347
Quantity of nutrition claims	0.075	0.027	2.727	**0.006**
Understandability of nutrition claims	−0.020	0.030	−0.662	0.508
Importance of nutrition claims	0.225	0.031	7.284	**<0.001**
Credibility of nutrition claims	0.048	0.031	1.554	0.121
Nutrition information at first purchase	0.025	0.026	0.952	0.341
Willingness to pay for nutrition claims	0.069	0.031	2.204	**0.028**
Attention to health and nutrition claims	0.144	0.035	4.111	**<0.001**
Unreliable nutrition claims	0.106	0.027	3.920	**<0.001**
Diet healthiness evaluation	0.074	0.034	2.206	**0.028**
Knowledge about healthy nutrition	0.066	0.033	2.011	**0.045**
Dietary supplements	−0.020	0.026	−0.769	0.442
Organic food	0.062	0.029	2.109	**0.035**
Functional food	0.001	0.028	0.033	0.973
Fair trade products	−0.023	0.028	−0.796	0.426
FOP label reading in the shop	−0.098	0.034	-2.892	**0.004**
BOP label reading in the shop	0.033	0.035	0.949	0.343
FOP label reading at home	0.095	0.036	2.657	**0.008**
BOP label reading at home	0.038	0.037	1.026	0.305

Note: the operationalization of the variables is included in Table 1; SE—standard error. Significant values are shown in bold.

**Table 6:** Selected predictors of the importance attached to salt content information on food packaging (the final multiple regression model).

Predictors	β	SE	t	*p*
Intercept	x	x	−0.570	0.569
Sex	0.059	0.027	2.174	**0.030**
Age	0.156	0.027	5.857	**<0.001**
Quantity of nutrition claims	0.074	0.026	2.819	**0.005**
Importance of nutrition claims	0.237	0.030	8.026	**<0.001**
Willingness to pay for nutrition claims	0.070	0.030	2.313	**0.021**
Attention to health and nutrition claims	0.161	0.034	4.755	**<0.001**
Unreliable nutrition claims	0.105	0.026	3.972	**<0.001**
Diet healthiness evaluation	0.080	0.032	2.500	**0.013**
Knowledge about healthy nutrition	0.066	0.032	2.076	**0.038**
Organic food	0.057	0.029	1.988	**0.047**
FOP label reading in the shop	−0.086	0.032	−2.638	**0.008**
FOP label reading at home	0.111	0.033	3.400	**0.001**

Note: the operationalization of the variables is included in Table 1; SE—standard error. Significant values are shown in bold.

**Table 7:** ISCI predicted for different levels of independent variables in the final multiple regression model.

Predictors	Predictor Levels	ISCI Estimate	95% CI
Sex	Men	3.710	3.633–3.788
	Women	3.831	3.758–3.903
Age	20 years old	3.546	3.454–3.638
	30 years old	3.637	3.568–3.706
	40 years old	3.729	3.675–3.782
	50 years old	3.820	3.766–3.874
	60 years old	3.911	3.842–3.980
Quantity of nutrition claims	Excessive	3.612	3.487–3.736
	Appropriate	3.748	3.693–3.803
	Insufficient	3.884	3.792–3.976
Importance of nutrition claims	None	3.064	2.883–3.245
	Rather small	3.337	3.218–3.456
	Average	3.610	3.545–3.676
	Rather big	3.883	3.825–3.942
	Very big	4.156	4.050–4.263
Willingness to pay for nutrition claims	Definitely not	3.612	3.464–3.759
	Rather not	3.682	3.587–3.776
	Hard to say	3.751	3.696–3.807
	Rather yes	3.821	3.756–3.886
	Definitively yes	3.891	3.779–4.002
Attention to health and nutrition claims	Definitely not	3.327	3.134–3.518
	Rather not	3.508	3.386–3.629
	Hard to say	3.689	3.627–3.752
	Rather yes	3.871	3.806–3.936
	Definitively yes	4.052	3.927–4.178
Unreliable nutrition claims	Definitely not	3.415	3.229–3.600
	Rather not	3.539	3.412–3.666
	Hard to say	3.664	3.588–3.739
	Rather yes	3.788	3.736–3.840
	Definitively yes	3.912	3.827–3.998
Diet healthiness evaluation	Very unhealthy	3.491	3.263–3.720
	Rather unhealthy	3.612	3.475–3.750
	Average	3.733	3.673–3.794
	Rather healthy	3.854	3.773–3.936
	Very healthy	3.975	3.809–4.141
Knowledge about healthy nutrition	Very small	3.549	3.329–3.768
	Rather small	3.645	3.511–3.778
	Average	3.741	3.680–3.801
	Rather big	3.836	3.758–3.915
	Very big	3.932	3.774–4.090
Organic food	Purchasing	3.828	3.754–3.903
	Not purchasing	3.711	3.630–3.792
FOP label reading in the shop	10% of food products	3.919	3.800–4.038
	50% of food products	3.792	3.738–3.845
	90% of food products	3.664	3.567–3.761
FOP label reading at home	10% of food products	3.629	3.530–3.727
	50% of food products	3.778	3.726–3.830
	90% of food products	3.927	3.825–4.030

Notes: the operationalization of the variables is included in Table 1; CI—confidence interval.
